# Quality of life profiles and their association with clinical and demographic characteristics and physical activity in people with a stoma: a latent profile analysis

**DOI:** 10.1007/s11136-022-03102-5

**Published:** 2022-02-25

**Authors:** William Goodman, Amy Downing, Matthew Allsop, Julie Munro, Claire Taylor, Gill Hubbard, Rebecca J. Beeken

**Affiliations:** 1 School of Medicine, University of Leeds, Clarendon Way, Leeds, LS2 9JT UK; 2 Department of Nursing, University of the Highlands and Islands, 12b Ness Walk, Inverness, IV3 5SQ UK; 3 London North West Healthcare NHS Trust, Watford Road, Harrow, HA1 3UJ UK; 4 Research Department of Behavioural Science and Health, University College London, Gower Street, London, WC1E 6BT UK

**Keywords:** Quality of life, Stoma, Hernia, Physical activity

## Abstract

**Purpose:**

Quality of life can be negatively impacted by the formation of a stoma and is influenced by a number of factors. Research to date treats people with a stoma as a homogenous group based on their quality of life. We attempted to identify subgroups based upon self-reported quality of life and explored variables associated with group membership.

**Methods:**

The present study is a secondary analysis of a cross-sectional sample of 1419 people with a stoma. Participants completed validated questionnaires for quality of life, physical activity and clinical and demographic characteristics. Latent profile analysis was used to identify the optimal number of subgroups (profiles) and multinomial regression modelling was conducted to identify variables associated with profile membership.

**Results:**

The analysis revealed 4 distinct profiles of people with a stoma: ‘consistently good quality of life’ [*N* = 891 (62.8%)], ‘some quality of life concerns’ [*N* = 184 (13.0%)], ‘low quality of life’ [*N* = 181 (12.8%)] and ‘financial concerns’ [*N* = 163 (11.5%)]. Modelling revealed that people with a recent stoma (formed < 2 years previously), who have a hernia and are less physically active were more likely to belong to the ‘low quality of life’ profile. Furthermore, those aged 16–55 were more likely to have financial concerns.

**Conclusion:**

This study was the first to identify latent profiles within this population and assess whether certain variables are associated with membership. Future research should build upon this to identify additional variables associated with these profiles, which can help to provide the basis for targeting and tailoring future interventions to specific subgroups of people with a stoma.

**Supplementary Information:**

The online version contains supplementary material available at 10.1007/s11136-022-03102-5.

## Background

A bowel stoma is an artificial opening on the abdomen that has been created to divert the flow of faeces [1]. There are no current global estimates for the number of people living with a stoma; however, available estimates from the USA and China suggest that there are about 1 million people with a stoma in each country and around 700,000 people with a stoma in Europe [2, 3]. Colorectal cancer is thought to be the foremost cause for creation of a stoma with data from the UK and Sweden suggesting that 25–32% of patients who undergo surgery will have a stoma formed [4, 5]. Stomas can also be formed due to inflammatory bowel disease (IBD), diverticular disease, physical trauma and incontinence [1]. Quality of life (QoL) can be impaired in patients post stoma formation surgery. Research amongst Swedish rectal cancer patients found that those without a stoma had higher levels of QoL compared to those with a stoma [6]. However, a study of Crohn’s Disease patients with and without a stoma found no difference in overall QoL but did find lower social role satisfaction in those with a stoma [7]. Additional research has also indicated that this difference in QoL between those with and without a stoma can remain for over 2–5 years [8, 9].

QoL in people with a stoma can be impacted by a number of different stoma-related problems [10], which are captured by stoma-specific QoL measures such as the Stoma Quality of Life Scale that cover work and social issues, body image concerns, issues with the stoma and financial matters [11]. These problems may be influenced by demographic and clinical factors such as age, gender and time since treatment. Clinical characteristics including presence of a hernia, cancer being the underlying disease and having an ileostomy compared to having a colostomy can also be associated with lower levels of QoL [12–15]. Additionally, there is a growing body of evidence to suggest that behaviours such as being physically active can have an important effect on QOL in colorectal cancer survivors and people with IBD [16, 17].

The current research into QoL amongst people with a stoma has looked at the variables that are associated with QoL; however, there has been no investigation as to whether there are distinct subgroups of people with a stoma who report specific patterns of QoL. Latent profile analysis (LPA) takes a person-oriented approach by identifying subpopulations within the sample based upon responses to certain variables [18]. This method lends itself to the multidimensional nature of QoL by allowing us to identify whether there are distinct groups of people with similar patterns of QoL responses. This can allow for identification of those who are most in need of intervention or may need intervention in different areas, which is in line with the recent development of a person-centred approach to healthcare [19, 20]. This method has been used previously to identify areas for future development of behaviour change interventions for diet [21], sexual health [22] and alcohol and drug problems [23] and also interventions for patients with hypertension [24] and for older adults [25]. For example, a study by Choi et al. [25] identified four distinct profiles (physical disability type, emotional disability type, crisis type and stable type) based upon health-related QoL scores amongst older adults (over 65 years). They found that membership of people in the ‘emotional disability’ profile, who were characterised by low scores on the anxiety and depression subscale but higher scores on the other subscales, was associated with lower scores on happiness, depression and cognitive decline scales and belonging to a one-person household compared to the ‘stable’ profile. They concluded that this profile would benefit from an intervention aimed at mental health assistance. Therefore, using latent profile analysis within a sample of people with a stoma may help us to identify how to better tailor interventions.

The primary purpose of this study was to identify distinct subpopulations of individuals with a stoma based upon their self-reported QoL. We also assessed whether membership of these groups was associated with demographic and clinical characteristics and physical activity (PA). To our knowledge no previous research has conducted this type of analysis within this population, consequently no hypotheses were formed due to the exploratory nature of the study.

## Methods

### Study design

The present study was a secondary analysis of data from a cross-sectional, observational survey conducted between 26 April and 16 May 2018. This was an exploratory survey investigating the relationship between support garments and stoma-related QoL. Ethical approval for the original study was obtained from the University of the Highlands and Islands Research and Ethics Committee (Ref: OLETHSHE903), and approval to use this data for the present study was obtained from the lead investigator of the original study, GH. See Online Resource 1 for a full list of the variables within the original dataset.

### Participants

A sample of 1528 participants was obtained using a convenience sampling method. The total number of people contacted is not known. Participants were asked to complete the survey if they currently had, or had ever had, at least one type of stoma (ileostomy, colostomy or urostomy), were at least 16 years old and could answer questions in English. For the present study, the focus was on people with a bowel stoma; therefore, those with a urostomy or who did not select any stoma were removed leaving a final sample of 1419. The sample size was determined to be appropriate for this study based on a rule of thumb established in previous latent profile analysis studies. A Monte Carlo simulation study looked at a number of different sample sizes and concluded that a sample size of 500 would be sufficient to identify the optimal number of profiles [26].

### Procedure

The survey was hosted on the Online Surveys website. The link to the survey was distributed via social media (Twitter and Facebook) and through an email sent to customers of Vanilla Blush, a UK-based stoma and hernia support garment supplier. Participants were directed to a page that gave them information on the survey and its aims and were asked to consent by ticking a box.

For the present study members of our stakeholder group were approached to consult on the cut-offs that were used for the variables of age, number of abdominal surgeries and time with a stoma. Two people with a stoma, a charity representative and two stoma nurse specialists were members of a stakeholder group formed to provide advice and feedback on a body of research related to people with a stoma. All contact with members of the stakeholder group was via email or teleconference.

### Measures

#### Demographic and clinical characteristics

Sex was originally measured as ‘Female’, ‘Male’ and ‘Other’, but due to the small number of ‘Other’ (*N* = 3) these were set to missing and a dichotomous variable was created (Male, Female). Age was measured as age range [8-point scale(16–25 to 86 +)]. For the purpose of this study this ordinal variable was dichotomised into aged 55 or younger and aged 56 +. This was based on a review of the distribution of the data and feedback on the appropriateness of the cut-off from the stakeholder group. The reasoning given from the stakeholder group was that those who were below the age of 55 were more likely to have IBD and those older were more likely to have cancer.

The presence of a hernia or bulge was measured by three questions; if they had ever had a medically diagnosed parastomal or incisional hernia and whether they had a bulge around their stoma. These were combined and dichotomised into either ‘No’ hernia or bulge or ‘Yes’ hernia or bulge. The reason for stoma formation was selected from IBD, Cancer, Physical Trauma or Other. Some of the other reasons that were outlined by participants included diverticulitis, familial adenomatous polyposis (FAP) and Hirschsprung’s disease. Participants selected their type of stoma between ileostomy and colostomy. The number of abdominal surgeries [4-point scale (1–4 or more surgeries)] and the range of time with a stoma [7-point scale (0–6 months to more than 4 years)] were both measured as ordinal variables. For the purpose of this study these variables were dichotomised into: abdominal surgeries 1 or 2 + and time with a stoma ≤ 2 years or > 2 years. These decisions were also based on a review of the distribution of the data and feedback on the appropriateness of the cut-offs from the stakeholder group. The reasoning given by the stakeholder group for these cut-offs was that for abdominal surgeries it was felt that those having multiple surgeries would have a different experience than those that only required 1 surgery and for the time with a stoma at 2 years would be sufficient to capture those that were learning to manage their stoma.

#### Physical activity

PA was measured using an adapted single-item tool [27]. Respondents rated on a scale how many days in the past week they had done 30 min or more PA that raised their breathing rate, this was measured from ‘0 days’ to ‘7 days’.

#### Quality of Life

QoL was measured by the Stoma Quality of Life Scale (SQoL) [11]. The SQoL contains 19 items over 5 subscales: Work/Social Function, Sexuality and Body Image, Stoma Function, Financial Concerns and Skin Irritation; these are measured on a 5-point Likert scale from ‘Never’ to ‘Always’. The results for each subscale are then transformed into a 0–100 scale based upon the algorithm in Baxter et al. [11]. The reliability of the overall scale is rated as good (α = .89).

### Statistical analysis

Statistical analyses were conducted using IBM SPSS v26 and Latent GOLD v5.1. Descriptive statistics were run on all variables included.

Within the data, 2.3% (898 of 37,415) of values were missing but 26.4% (375 of 1419) of cases had a single missing data point. The variables with the most missing data points were two items on the sexuality and body image subscale [‘My sexual partner is bothered by my stoma’, *N* = 191 (13.5%); ‘I enjoy sexual activity’, *N* = 190 (13.4%)] and the single item on the financial concerns subscale [‘I have financial concerns regarding my stoma supplies’, *N* = 137 (9.7%)]. Little’s MCAR test was run to determine whether the data were missing completely at random or not. The MCAR test was significant [χ^2^(1108) = 1505.9, *p* < .001]; therefore, the data were not missing completely at random. To account for this, the LPA was run using the maximum likelihood method which uses all data available.

A three-step approach was taken to conducting the LPA. The first step identified the appropriate number of profiles based upon responses to the SQoL subscales. Initially a single profile was run with this increasing to 5 (the number of subscales of SQoL). The models were then compared across multiple indicators of model fit: Akaike Information Criteria (AIC), Bayesian Information Criteria (BIC) and entropy. For AIC and BIC a lower number indicates better model fit whereas for entropy a number closer to 1.00 indicates better latent profile separation. However, as there is no gold standard for model fit statistics for LPA, the models were also evaluated based upon their interpretability, and models with groups of 5% of the sample or smaller were rejected. The second step involved assigning participants to a profile based upon their probability scores. A one-way analysis of variance (ANOVA) test was conducted to determine whether there were differences in the QoL subscales mean scores across the profiles. Post-hoc Bonferroni tests were run to assess mean differences between each profile. The final step involved running a multinomial regression to assess whether there was a difference in profile membership based upon demographics, clinical characteristics and PA. This was run with the maximum likelihood method to account for potential bias in classification errors and non-random missing data [28]. Overall differences across profiles on each variable were assessed by running Omnibus Wald tests, with Wald Χ^2^ pairwise comparison tests being run to test for differences for each variable between profiles, the Bonferroni correction for multiple analyses was applied.

## Results

### Descriptive statistics

Table 1 provides an overview of the demographic and clinical characteristics of the sample alongside the mean scores for QoL. The sample of 1419 ostomates was predominantly female (79.1%), had an ileostomy (67.4%) and had their stoma formed because of IBD (55.3%). Just under half of the sample reported having a hernia or bulge (48.3%) and the mean reported number of days of PA per week was 2.6 (SD = 2.3). For the SQoL subscales [range 0 (low QoL)–100 (high QoL)] financial concerns had the highest mean score of 81.3(SD 28.5) and skin irritation had the lowest mean score of 47.2 (SD 27.9).

**Table 1:** Descriptive statistics for the sample (*N* = 1419)

Demographic and clinical characteristics	
Sex (*N*, %)	
Female	1122 (79.1)
Male	289 (20.4)
Missing	8 (0.6)
Age (*N*, %)	
16–55	961 (67.7)
56 +	451 (32.2)
Missing	1 (0.1)
Stoma (*N*, %)	
Ileostomy	956 (67.4)
Colostomy	444 (31.3)
Missing	19 (1.3)
Reason for stoma formation (*N*, %)	
IBD	785 (55.3)
Cancer	328 (23.1)
Physical trauma	103 (7.3)
Other	188 (13.2)
Missing	15 (1.1)
Hernia or bulge (*N*, %)	
No	727 (51.2)
Yes	685 (48.3)
Missing	7 (0.5)
Time with stoma (*N*, %)	
0–24 months	479 (33.8)
More than 2 years	926 (65.3)
Missing	14 (1.0)
Number of abdominal surgeries (*N*, %)	
1	335 (23.6)
2 or more	304 (21.4)
Missing	18 (1.3)
Stoma Quality of Life subscales	
Work and social function (M, SD)	63.6 (23.0)
Missing (*N*, %)	57 (4.0)
Sexuality/body image (M, SD)	61.5 (19.3)
Missing (*N*, %)	198 (14.0)
Stoma function (M, SD)	52.8 (20.6)
Missing (*N*, %)	16 (1.1)
Financial concerns (M, SD)	81.3 (28.5)
Missing (*N*, %)	137 (9.7)
Skin irritation (M, SD)	47.2 (27.9)
Missing (*N*, %)	13 (0.9)
Physical activity	
No. days per week (M, SD)	2.6 (2.3)
Missing (*N*, %)	5 (0.4)

*N* number of participants, *M* mean, *SD* standard deviation, *IBD* Inflammatory Bowel Disease

### Latent profile analysis

The model fit statistics for the five LPA models are outlined in Table 2. AIC and BIC decreased with the addition of each additional latent profile. Entropy decreased initially with the addition of a latent profile but began to increase from the four latent profile model. The five-profile model had a profile with only 4.8% of the sample in and so was rejected. Based upon these statistics and the interpretability of the model a four-profile model was selected.

**Table 2:** Model fit statistics

Number of profiles	AIC	BIC	Entropy	Smallest profile %
1	61156.1	61208.7	1.00	NA
2	55916.7	56027.1	0.90	38.7
3	55160.1	55328.4	0.84	18.0
4	54186.3	54412.4	0.88	11.5
5	53196.6	53480.5	0.89	4.8

*AIC* Akaike Information Criteria, *BIC* Bayesian Information Criteria

**Fig. 1:**
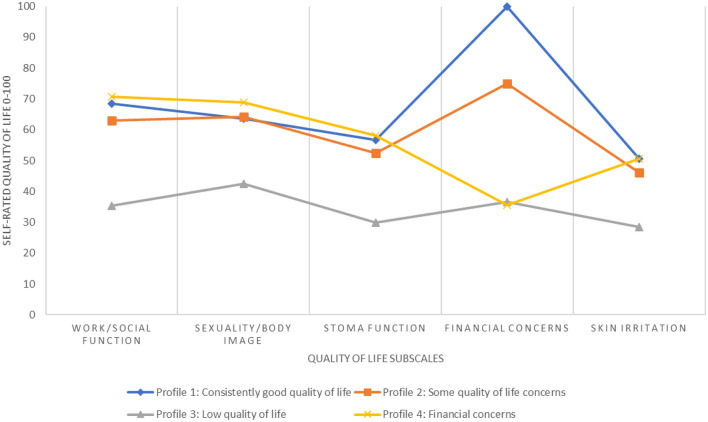
Quality of life subscales for the latent profile classes

Table 3 presents the estimated mean SQoL scores from the 4-profile model and Fig. 1 plots this. One-way ANOVA tests indicate there were significant differences between the profiles for all the SQoL subscales and the post-hoc Bonferroni tests indicate which profiles were different to each other. Profile 1 (N = 891, 62.8%) was characterised by a high score on financial concerns but also higher than average QoL scores across all subscales and so was labelled ‘consistently good quality of life’. Profile 2 (N = 184, 13.0%) was characterised by moderate QoL concerns across all subscales and was labelled ‘some quality of life concerns’. Profile 3 (N = 181, 12.8%) was characterised by low QoL scores across all the subscales and was labelled ‘low quality of life’. Profile 4 (N = 163, 11.5%) was characterised by its low score on financial concerns but high scores on work/social function and sexuality/body image and was labelled ‘financial concerns’.

**Table 3:** Final class count and proportions, and quality of life scores for each profile

	Profile 1—Consistently good quality of life *N* = 891 (62.8%)	Profile 2—Some quality of life concerns *N* = 184 (13.0%)	Profile 3—Low quality of life *N* = 181 (12.8%)	Profile 4—Financial concerns *N* = 163 (11.5%)	*p* value	Bonferroni post-hoc test
Posterior probabilities Mean (SD)	0.96 (0.11)	0.97 (0.06)	0.89 (0.15)	0.94 (0.11)	–	–
Quality of life scores: mean (SD)						
Work/social function	*68.4 (21.7)*	**63.0 (19.8)**	**35.3 (15.0)**	*70.6 (15.9)*	< .001	1 > 2,3; 2 > 3; 2 < 4, 3 < 4
Sexuality/body image	*63.5 (19.0)*	*64.1 (17.4)*	**42.4 (12.8)**	*68.9 (15.9)*	< .001	1 > 3; 1 < 4; 2 > 3; 3 < 4
Stoma function	*56.6 (20.1)*	**52.4 (17.8)**	**30.0 (14.1)**	*58.1 (15.4)*	< .001	1 > 2,3; 2 > 3; 2 < 4; 3 < 4
Financial concerns	*100.0 (*< *0.1)*	**75.0 (< 0.1)**	**36.5 (22.6)**	**35.6 (20.2)**	< .001	1 > 2,3,4; 2 > 3,4
Skin irritation	*50.7 (28.2)*	**46.2 (25.1)**	**28.5 (23.1)**	*50.5 (26.0)*	< .001	1 > 3; 2 > 3; 3 < 4

Bonferroni post-hoc tests difference at p < .05 between each class on each subscale (1 = Consistently good quality of life, 2 = Some quality of life concerns, 3 = Low quality of life, 4 = Financial concerns), e.g. for Work/social function ‘1 > 2,3’ means that profile 1 has a mean score that is larger than profiles 2 and 3 and this is statistically significant at *p* < .05

Highlighted italics—above the quality of life subscale mean, highlighted bold—below the quality of life subscale mean

*N* Number of participants, *SD* standard deviation

### Association with latent profile membership

Table 4 outlines the clinical and demographic characteristics and PA of the membership of each profile. This table also shows Omnibus Wald tests which indicated that there were overall differences between profiles on the reason for the stoma formation, age of the individual, the presence of a hernia or bulge and the PA of the individual (*p* < 0.05). Wald Χ^2^ pairwise comparison tests identify that Profile 3 (‘Low quality of life’) is different from the other three profiles with a greater proportion of people having a hernia or bulge in this profile than the other three. Furthermore, individuals classified into profile three were on average spending 1 less day being physically active than those in the other profiles.

**Table 4:** Characteristics of each latent profile

N (%)	Profile 1—Consistently good quality of life (*N* = 891)	Profile 2—Some quality of life concerns (*N* = 184)	Profile 3—Low quality of life (*N* = 181)	Profile 4—Financial concerns (*N* = 163)	Wald Omnibus *p* value
Reason for stoma formation		^3^	^2^		0.006
IBD	475 (53.3)	118 (64.1)	88 (48.6)	104 (63.8)	
Cancer	232 (26.0)	33 (17.9)	32 (17.7)	31 (19.0)	
Physical trauma	65 (7.3)	14 (7.6)	14 (7.7)	10 (6.1)	
Other	111 (12.5)	16 (8.7)	45 (24.9)	16 (9.8)	
Stoma					0.096
Ileostomy	599 (67.2)	126 (68.5)	115 (63.5)	116 (71.2)	
Colostomy	281 (31.5)	54 (29.3)	63 (34.8)	46 (28.2)	
Time with stoma					0.078
2 years or less	294 (33.0)	67 (36.4)	71 (39.2)	47 (28.8)	
More than 2 years	589 (66.1)	114 (62.0)	110 (60.8)	113 (69.3)	
Number of abdominal surgeries					0.16
1	221 (24.8)	40 (21.7)	30 (16.6)	44 (27.0)	
2 +	657 (73.7)	144 (78.3)	150 (82.9)	115 (70.6)	
Sex					0.41
Female	697 (78.2)	144 (78.3)	154 (85.1)	127 (77.9)	
Male	191 (21.4)	37 (20.1)	27 (14.9)	34 (20.9)	
Age	^2^	^1^			0.006
16–55	568 (63.7)	144 (78.3)	127 (70.2)	122 (74.8)	
56 +	322 (36.1)	40 (21.7)	54 (29.8)	41 (25.2)	
Hernia	^3^	^3^	^1,2,4^	^3^	< .001
No	479 (53.8)	110 (59.8)	54 (29.8)	84 (51.5)	
Yes	407 (45.7)	74 (40.2)	125 (69.1)	79 (48.5)	
Mean (SD)					
Physical activity (days)	2.7 (2.3)^3^	2.9 (2.3)^3^	1.9 (2.1)^1,2,4^	2.9 (2.3)^3^	0.001

Superscript numbers relate to Wald Χ^2^ pairwise comparison tests at *p* < .05 between each class and the class number indicated (1 = Consistently good quality of life, 2 = Some quality of life concerns, 3 = Low quality of life, 4 = Financial concerns)

*IBD* Inflammatory Bowel Disease, percentages might not add up to 100% due to missing data

Table 5 outlines the results of the multinomial regression investigating the association between these characteristics and membership of a specific profile, with Profile 1 being used as the reference category within the analysis. Individuals classified into profile 2 (‘Some quality of life concerns’) and profile 4 (‘Financial concerns’) were younger compared to profile 1. Those in profile 2 were more likely to have a colostomy (OR 1.64, 95%CI 1.08, 2.49) and were less likely to have their stoma formed because of ‘cancer’ (OR 0.57, 95%CI 0.35, 0.93) or ‘other’ reasons (e.g. Diverticulitis, FAP and Hirschsprung’s Disease) (OR 0.52, 95%CI 0.28, 0.98).

**Table 5:** Variables associated with profile membership

	Profile 2—Some quality of life concerns	Profile 3—Low quality of life	Profile 4—Financial concerns
Odds ratio (95% CI)			
Reason for stoma formation (reference: IBD)			
Cancer	**0.57 (0.35; 0.93)**	0.76 (0.41; 1.41)	0.60 (0.34; 1.08)
Physical trauma	0.86 (0.44; 1.68)	0.87 (0.42; 1.78)	0.67 (0.31; 1.48)
Other	**0.52 (0.28; 0.98)**	**1.86 (1.10; 3.15)**	0.57 (0.27; 1.18)
Stoma (reference: ileostomy)			
Colostomy	**1.64 (1.08; 2.49)**	1.20 (0.72; 2.00)	1.40 (0.83; 2.35)
Time with stoma (reference: 2 years or less)			
More than 2 years	0.81 (0.56; 1.16)	**0.65 (0.43; 0.96)**	1.22 (0.80; 1.85)
Number of abdominal surgeries (reference: 1)			
2 +	1.27 (0.83; 1.93)	1.37 (0.85; 2.22)	0.78 (0.53; 1.16)
Sex (reference: female)			
Male	1.02 (0.66; 1.56)	0.64 (0.38; 1.08)	0.96 (0.62; 1.50)
Age (reference: 16–55)			
56 +	**0.56 (0.37; 0.85)**	0.66 (0.42; 1.02)	**0.64 (0.42; 0.98)**
Hernia (reference: no)			
Yes	0.93 (0.65; 1.33)	**3.32 (2.17; 5.07)**	1.26 (0.87; 1.81)
Per day increase in physical activity	1.04 (0.97; 1.12)	**0.85 (0.78; 0.94)**	1.07 (0.99; 1.15)

Bold values indicate statistical significance at *p* < .05

Profile 1 ‘Consistently good quality of life’ is the reference category

*CI* Confidence Interval, *IBD* Inflammatory Bowel Disease

Those classified into profile 3 (‘Low quality of life’) were less likely to have had their stoma for longer than 2 years (OR 0.65, 95%CI 0.43, 0.96) and to spend more days being physically active (OR 0.85, 95%CI 0.78, 0.94) but were more likely have a hernia or bulge (OR 3.32, 95%CI 2.17, 5.07).

## Discussion

This study is the first to identify that people with a stoma are heterogenous in how they report their QoL. Four distinct profiles were identified, with ‘consistently good quality of life’ being the most common and ‘some quality of life concerns’, ‘low quality of life’ and ‘financial concerns’ being of roughly equal size. The results of this study suggest that members of all profiles could benefit from additional support around social and work situations, body image concerns and how to deal with stoma function issues and skin irritation. However, a more intensive intervention may be required for those who have recently had a stoma, have a hernia or have had their stoma formed for ‘other’ reasons (e.g. diverticulitis, FAP and Hirschsprung’s Disease) as these individuals were more likely to belong to the ‘low quality of life’ profile according to our regression analyses. Furthermore, those who were less physically active were also more likely to belong to this profile, which could be a consequence of their clinical characteristics but could suggest that they may benefit from an intervention encouraging PA. To the best of our knowledge this is the first study to identify the QoL profiles of people with a stoma and to explore the factors associated with membership of these profiles. These findings provide us with a basis on which to tailor interventions to those most in need.

The results of the regression indicate that age may play a role in financial concerns. Those who are older and possibly retired may feel more secure in their financial position, compared to those who are younger and who, therefore, may have more concerns about the impact of their stoma on their working/financial situation. Previous qualitative work has highlighted that some people with a stoma have concerns about their working situation, and some do not return to work post-surgery [29]. This might explain why younger individuals are more likely to belong to profiles 2 ‘some quality of life concerns’ and 4 ‘financial concerns’ and might, therefore, benefit from more support and information on returning to work and managing financial difficulties. However, this may also be dependent on geographical location, as countries may have different levels of generosity of social security for older people and even within countries different health authorities may provide varying levels of support for people with a stoma.

This study identified one profile that had consistently lower QoL scores across all areas. The’low quality of life’ profile accounted for 12.8% of the sample and included individuals who were more likely to have a recent stoma and the stoma formed for ‘other’ reasons. This is in line with previous research; a small (*N* = 49) prospective study of patients with a stoma found that QoL improved over time with younger patients [30]. Two cross-sectional studies have found an association between QoL and self-efficacy in people with a stoma [31, 32], which could suggest that as their confidence in managing their stoma improves so would their QoL. However, research is needed with prospective cohorts to determine whether, as people progress with their stoma, they transition from the ‘low quality of life’ profile to one with improved QoL. There is also currently little research on QoL in people with a stoma beyond those that have had a stoma formed due to cancer or IBD. Further research is needed in other disease areas to unpick the finding that those with a stoma formed for ‘other’ reasons are more likely to be in the ‘low quality of life’ profile. Available services to support people with a stoma post-surgery may currently be more relevant to those with IBD and cancer than to those from less common diseases.

Individuals classified into profile 3 ‘low quality of life’ were also more likely to have a hernia or bulge and were less physically active. These findings are in line with previous cross-sectional research which suggests that the presence of a hernia or bulge is associated with lower QoL scores [6, 33, 34]. Further cross-sectional research also suggests that the presence of a bulge or hernia is associated with lower levels of PA [6]. However, these relationships need to be modelled over time to determine causality. Interventions that target these issues could improve QoL within this profile. For example, the Hernia Active Living Trial [35], which is recruiting people with a stoma and a hernia or bulge, is seeking to improve QoL and physical fitness through strengthening the abdominal wall to reduce hernia progression.

There may be additional factors associated with membership of the profiles that were not measured. For example, complications with the stoma or other health issues may have been associated with the ‘low quality of life’ profile [2, 36, 37]. Interestingly, we did not find an association between membership of this profile and the type of stoma. Previous research has suggested that 22–35% of people with an ileostomy report a daytime leakage compared to 12–20% of people with a colostomy with similar results reported for night-time leakages (ileostomy 21–33% vs colostomy 3–15%), which could contribute to lower QoL [14]. Furthermore, variables such as health services use, health outcomes and engagement with offered interventions or support would be useful to know to understand the level of support required. Additional research is required to assess whether other factors may be associated with membership of the profiles to develop a more comprehensive view on interventions that could benefit this group. Research will also need to consider how future interventions will be utilised by health professionals to target those individuals in need within health services.

Strengths of this study are the large sample size and the focus on a person-centred approach by using LPA to identify profiles of QoL responses. The LPA approach has produced profiles that provide suggestions for future tailored interventions. Further exploratory cross-sectional studies are needed to confirm and expand upon the findings of this study. However, cross-sectional studies can only examine associations. Further research that can take a longitudinal perspective and explore the dynamic interaction of QoL over the course of an individual’s life is needed, using latent transition analysis, for example. Furthermore, future research could strengthen the identification of the profiles by partitioning samples into training and validation sets to run profile identification and then validate the findings.

There are limitations with the present study. Firstly, the sample may not be representative of the wider stoma population as the majority of the sample had their stoma formed because of IBD when cancer is the most common reason for stoma formation [4]. This could be due to the method of recruitment as social media and the mailing list of a support garment supplier were used, which may have biased the sample towards younger age groups who are more likely to have had IBD. As this study was a secondary analysis of previously collected data, the sampling methods used, and the variables collected were not optimised for the aims of the present study. For example, certain variables were not ideal (e.g. age range instead of age, which reduces the precision of the variable; single item of PA instead of time spent over a week; and the Stoma QoL Scale which requires further validation) and other variables such as BMI, which is associated with higher rates of stoma complications [38] were not available. Variables that were used within the analysis may also overlap, such as age and reason for stoma formation; however, tests for multicollinearity indicated only moderate overlap. Also, although we identified different profiles based upon reported QoL we do not know whether the differences between profiles, although statistically significant, are clinically meaningful. Future research should consider employing the Delphi technique to help identify what the minimum clinically meaningful difference would be on these scales.

In conclusion, this is the first study to identify latent profiles within a sample of people with a stoma and highlights that the sample is heterogenous in how they report QoL. Furthermore, it suggests that different groups may benefit from different interventions or support. For example, those who have a recent stoma, a hernia, are less physically active or have had their stoma formed for ‘other’ reasons may benefit from more intensive support as they are more likely to have inhibited QoL. Additionally, those who are younger may benefit from additional support around financial issues and advice on returning to work. Future research is required to explore the consistency of these profiles across more representative samples and to expand the range of variables associated with profile membership. Further work in this area will improve the development and tailoring of interventions to enhance QoL for people living with a stoma.

## Supplementary Information

Below is the link to the electronic supplementary material.

Supplementary file1 (DOCX 15 KB)

## Data Availability

This was secondary data analysis.
