# Supplementary material for: Quality of life profiles and their association with clinical and demographic characteristics and physical activity in people with a stoma: a latent profile analysis
Source: Qual Life Res. 2022 Feb 25;31(8):2435–44. doi: 10.1007/s11136-022-03102-5 (PMC9250477; doi:10.1007/s11136-022-03102-5)
Supplement: Supplementary file 1 — Supplementary file1 (DOCX 15 KB) [file 11136_2022_3102_MOESM1_ESM.docx]

**Supplementary Material 1 – Full variable list**

Why did you have your stoma(s) formed?

If you selected Other, please specify:

COLOSTOMY Please select what type(s) of stoma you have (Please tick all that apply).

ILEOSTOMY Please select what type(s) of stoma you have (Please tick all that apply).

UROSTOMY Please select what type(s) of stoma you have (Please tick all that apply).

UNSURE Please select what type(s) of stoma you have (Please tick all that apply).

How long have you been living with a stoma?

How many abdominal surgeries have you had in your lifetime? (please include stoma surgery, and all non-stoma related surgery)

OPEN What types of abdominal surgery have you had? (including stoma surgery, and any non-stoma related surgery) (Please select ALL that apply)

LAPARASCOPIC What types of abdominal surgery have you had? (including stoma surgery, and any non-stoma related surgery) (Please select ALL that apply)

UNSURE What types of abdominal surgery have you had? (including stoma surgery, and any non-stoma related surgery) (Please select ALL that apply)

What age were you when you had your stoma?

Do you currently live in the UK?

If NO, please select which country you currently live in:

What is your sex?

What age are you?

Have you ever been told by a nurse or doctor that you've had/have a parastomal hernia?

Do you have/have you ever had a bulge around the stoma, making one side stick out a bit more?

Have you ever been told by a nurse or doctor that you've had/have an incisional hernia?

Has anyone discussed the different types of support garments with you?

STOMA NURSE If Yes, who discussed these with you? (Please select ALL that apply)

OTHER NURSE If Yes, who discussed these with you? (Please select ALL that apply)

MY GP If Yes, who discussed these with you? (Please select ALL that apply)

MY SURGEON If Yes, who discussed these with you? (Please select ALL that apply)

OTHER PERSON WITH STOMA If Yes, who discussed these with you? (Please select ALL that apply)

FAMILY MEMBER If Yes, who discussed these with you? (Please select ALL that apply)

SUPPLIER If Yes, who discussed these with you? (Please select ALL that apply)

SUPPORT GROUP If Yes, who discussed these with you? (Please select ALL that apply)

CHARITY If Yes, who discussed these with you? (Please select ALL that apply)

OTHER If Yes, who discussed these with you? (Please select ALL that apply)

If you selected Other, please specify:

Do you currently own any kind of support garment for your stoma? (including support belts/support underwear such as vests, pants, and girdles)

If you selected No, can you suggest a reason (didn't know about them? not interested?)

How many support BELTS do you currently own?

How many days have you worn your support BELT in the past month? (Leave blank if you do not own any)

How many support UNDERWEAR garments do you currently own (e.g. vests, pants, girdles)?

How many days have you worn your support UNDERWEAR in the previous month (including vests, pants, knickers, girdles)? (Leave blank if you do not own any)

How many support garments (belt or underwear) have you had on NHS prescription in the past year?

DIDN'T KNOW If you don't use your support garment(s) please select as many of the following reasons that may apply:

EXPENSIVE If you don't use your support garment(s) please select as many of the following reasons that may apply:

UNCOMFORTABLE If you don't use your support garment(s) please select as many of the following reasons that may apply:

NO BENEFIT If you don't use your support garment(s) please select as many of the following reasons that may apply:

VELCRO NOISY If you don't use your support garment(s) please select as many of the following reasons that may apply:

OTHER If you don't use your support garment(s) please select as many of the following reasons that may apply:

If you selected Other, please specify:

Rate your overall satisfaction with your life in general right now on a scale of 0 to 100. With 0 being totally unsatisfied and 100 being totally satisfied. Enter that number in the box below.

Rate your overall satisfaction with your life in general during the last month on a scale of 0 to 100, with 0 being totally unsatisfied and 100 being totally satisfied. Enter that number in the box below.

I am able to participate in hobbies that I enjoy

I am able to go out with friends

My stoma interferes with my ability to work or attend college/university/school

I worry about travelling because of my stoma

I enjoy sexual activity

I feel attractive

My sexual partner is bothered by my stoma

It bothers me if others are aware I have a stoma

I worry about lack of privacy when I need to empty my pouch

I feel comfortable in my clothing

I am satisfied with the foods I eat

I have financial concerns regarding my stoma supplies

I have problems with odour

I am able to share my feelings and concerns about my stoma with a family member or friend

I am embarrassed by gas (noises or rapid filling of bag)

I worry my stoma appliance will leak

I am bothered by skin irritation around the stoma

Social situations make me feel anxious

I perform the same household and family duties

It was difficult to hide the stoma appliance under clothing

I was self-conscious about the appearance of the stoma appliance

The stoma appliance limited the choice of clothes that I could wear

The stoma appliance was obvious to other people

The colour of the stoma appliance was discreet

It was difficult to hide the stoma appliance because of ballooning

The stoma appliance was comfortable to wear

I was not concerned about skin irritation under the stoma appliance (for example, feelings of burning, itching, pinching or pain)

It was uncomfortable to remove the stoma appliance from my body

I often forgot that I was wearing the stoma appliance

The stoma appliance was comfortable as it fitted well to my body movements

The stoma appliance disrupted my sleep during the night

In the past week on how many days have you done a TOTAL of 30 minutes or more of physical activity, which was enough to raise your breathing rate. This may include sport, exercise, and brisk walking or cycling for recreation or to get to and from places,

How confident are you that you can do gentle exercises to strengthen your abdominal muscles (range of motion, using weights, etc.)? - Not at all confident.........................Totally confident

How confident are you that you can do aerobic exercise such as walking, swimming, or bicycling? - Not at all confident.........................Totally confident

How confident are you that you can exercise without it causing problems with your stoma? - Not at all confident.........................Totally confident

How confident are you exercising WITHOUT your support garment? - Not at all confident.........................Totally confident

How confident are you exercising WITH your support garment? - Not at all confident.........................Totally confident

Please tell us anything you would like to add about your stoma, and your experience with support garments and/or belts.
